# Cleansing Blankets

**Published:** 1871-02

**Authors:** 


Cleansing Blankets.—It is quite as important to have the blankets on our beds clean as to have the sheets pure and white. The foul emanations which they absorb in time makes the bed anything but sweet. The Boston Journal of Chemistry gives the following method of cleansing blankets :

"Put two large teaspoonfuls of borax and a pint bowl of soft soap into a tub of cold water. When dissolved, put in a pair of blankets, and let them remain over night. Next day rub and drain them out, and rinse thoroughly in two waters, and hang them to dry. Do not wring them."

But this is not the only domestic use to which borax may be put. Says the same journal:

"Borax is the best cockroach exterminator yet discovered. This troublesome insect has a peculiar aversion to it, and will never return where it has once been scattered. As the salt is perfectly harmless to human beings, it is much to be preferred for this purpose to the poisonous substances commonly used.

"Borax is also valuable for laundry use, instead of soda. Add a handfull of it, powdered, to about ten gallons of boiling water, and you need use only half the ordinary allowance of soap. For laces, cambrics, etc., use an extra quantity of the powder. It will not injure the texture of the cloth in the least.

For cleansing the hair, nothing is better than a solution of borax water. Wash afterward with pure water, if it leaves the hair too stiff. Borax dissolved in water is also an excellent dentrifice, or tooth-wash.—*Herald of Health.*

